# Associations of Medicaid Expansion With Insurance Coverage, Stage at Diagnosis, and Treatment Among Patients With Genitourinary Malignant Neoplasms

**DOI:** 10.1001/jamanetworkopen.2021.7051

**Published:** 2021-05-19

**Authors:** Katharine F. Michel, Aleigha Spaulding, Ahmedin Jemal, K. Robin Yabroff, Daniel J. Lee, Xuesong Han

**Affiliations:** 1 University of Pennsylvania Perelman School of Medicine, Philadelphia; 2 Leonard Davis Institute of Health Economics, University of Pennsylvania, Philadelphia; 3 Surveillance and Health Equity Science, American Cancer Society, Atlanta, Georgia; 4 Department of Biostatistics and Epidemiology, College of Public Health, East Tennessee State University, Johnson City

## Abstract

**Importance:**

Health insurance coverage is associated with improved outcomes in patients with cancer. However, it is unknown whether Medicaid expansion through the Patient Protection and Affordable Care Act (ACA) was associated with improvements in the diagnosis and treatment of patients with genitourinary cancer.

**Objective:**

To assess the association of Medicaid expansion with health insurance status, stage at diagnosis, and receipt of treatment among nonelderly patients with newly diagnosed kidney, bladder, or prostate cancer.

**Design, Setting, and Participants:**

This case-control study included adults aged 18 to 64 years with a new primary diagnosis of kidney, bladder, or prostate cancer, selected from the National Cancer Database from January 1, 2011, to December 31, 2016. Patients in states that expanded Medicaid were the case group, and patients in nonexpansion states were the control group. Data were analyzed from January 2020 to March 2021.

**Exposures:**

State Medicaid expansion status.

**Main Outcomes and Measures:**

Insurance status, stage at diagnosis, and receipt of cancer and stage-specific treatments. Cases and controls were compared with difference-in-difference analyses.

**Results:**

Among a total of 340 552 patients with newly diagnosed genitourinary cancers, 94 033 (27.6%) had kidney cancer, 25 770 (7.6%) had bladder cancer, and 220 749 (64.8%) had prostate cancer. Medicaid expansion was associated with a net decrease in uninsured rate of 1.1 (95% CI, −1.4 to −0.8) percentage points across all incomes and a net decrease in the low-income population of 4.4 (95% CI, −5.7 to −3.0) percentage points compared with nonexpansion states. Expansion was also associated with a significant shift toward early-stage diagnosis in kidney cancer across all income levels (difference-in-difference, 1.4 [95% CI, 0.1 to 2.6] percentage points) and among individuals with low income (difference-in-difference, 4.6 [95% CI, 0.3 to 9.0] percentage points) and in prostate cancer among individuals with low income (difference-in-difference, 3.0 [95% CI, 0.3 to 5.7] percentage points). Additionally, there was a net increase associated with expansion compared with nonexpansion in receipt of active surveillance for low-risk prostate cancer of 4.1 (95% CI, 2.9 to 5.3) percentage points across incomes and 4.5 (95% CI, 0 to 9.0) percentage points among patients in low-income areas.

**Conclusions and Relevance:**

These findings suggest that Medicaid expansion was associated with decreases in uninsured status, increases in the proportion of kidney and prostate cancer diagnosed in an early stage, and higher rates of active surveillance in the appropriate, low-risk prostate cancer population. Associations were concentrated in population residing in low-income areas and reinforce the importance of improving access to care to all patients with cancer.

## Key Points

**Question:**

Is the Patient Protection and Affordable Care Act’s Medicaid expansion associated with the presentation and management of genitourinary cancers?

**Findings:**

In this case-control study including 340 552 patients with newly diagnosed genitourinary cancer in the National Cancer Database from 2011 to 2016, a difference-in-difference analysis found that, compared with states that did not expand Medicaid, Medicaid expansion was significantly associated with a decreased uninsured rate, an increased proportion of early-stage diagnosis for kidney and prostate cancers, and an increased proportion of patients receiving active surveillance for low-risk prostate cancer, with larger magnitudes of association observed in the low-income population.

**Meaning:**

These findings suggest that Medicaid expansion was associated with downstream diagnosis and treatment outcomes for genitourinary malignant neoplasms and may reduce socioeconomic disparities in these metrics.

## Teaser

This case-control study assesses the association of Medicaid expansion with health insurance status, stage at diagnosis, and receipt of treatment among patients with newly diagnosed genitourinary cancers.

## Introduction

One of the major components of the 2010 Patient Protection and Affordable Care Act (ACA) was the expansion of Medicaid coverage eligibility to 138% of the federal poverty level (FPL). While this expansion was intended to decrease rates of the individuals who are uninsured across the entire US population, in 2012, the Supreme Court made this expansion optional for states.^1^ In January 2014, 25 states and the District of Columbia opted to expand Medicaid, and several more states expanded in the ensuing years.^2^ This staggered and incomplete expansion pattern provides a natural experiment to study the association of the Medicaid expansion with population health.

The association of the Medicaid expansion with the detection and management of genitourinary malignant neoplasms is particularly important, since some of these cancers are among the most commonly diagnosed and costliest in the US. Prostate cancer is the most common cancer in men, and across sexes, bladder cancer is the sixth most common and kidney cancer is the eighth most common. Prostate, bladder, and kidney cancers collectively account for about 20% of newly diagnosed cancer cases in the US each year (347 080 of 1.8 billion estimated new cancer diagnoses in 2020).^3^ Regarding costs, prostate cancer is the fifth most expensive cancer, while bladder cancer is the ninth most expensive, and kidney cancer is the tenth most expensive, and these cancers accounted for more than $26 billion in estimated spending in 2020.^4^ Within these discussions of genitourinary cancer diagnosis and management, there are well-established racial/ethnic and socioeconomic disparities^5,6,7,8,9,10^ that could be potentially alleviated by expanded insurance coverage and access to care. Being uninsured is associated with higher odds of presenting with advanced stage cancer,^10,11,12,13,14,15,16^ being undertreated,^12,13,14,15^ and having worse survival.^10,12,14,15,17,18^ Furthermore, positive associations between health insurance coverage and outcomes are larger in magnitude for low-income populations.^19^

Previous research on the associations of Medicaid expansion with cancer care has focused on the association of expansion with the decreasing proportion of uninsured individuals rather than other aspects of cancer care, such as diagnosis and treatment.^20,21,22,23^ Only a handful of studies have studied further downstream metrics, and they have identified small shifts to earlier stage disease in a few nongenitourinary cancers^21,22,24^ and an increase in utilization of surgery for all cancers in aggregate.^25,26,27^ However, these studies have generally been limited to only a year of postimplementation data, and the association of Medicaid expansion with alleviating racial/ethnic or socioeconomic disparity has been inconsistent between different subgroups and cancer types.^20^ The objective of this study was to evaluate the association of Medicaid with the continuum of genitourinary cancer care, including insurance status, stage at diagnosis, and receipt of specific surgical and nonsurgical treatments, with a focus on patients residing in low-income areas.

## Methods

This case-control study was granted exemption from review by the Morehouse School of Medicine Institutional Review Board. Informed consent was waived because data were deidentified. This study is reported following the Reporting of Studies Conducted Using Observational Routinely-Collected Data (RECORD) reporting guidelines.

### Patient Population

Patients aged 18 to 64 years who were newly diagnosed with a first primary kidney, bladder, or prostate cancer between January 1, 2011, and December 31, 2016, were identified from the National Cancer Database (NCDB), a hospital-based cancer registry cosponsored by the American College of Surgeons and the American Cancer Society. The NCDB collects cancer diagnoses from all Commission on Cancer–accredited hospitals annually, capturing approximately 72% of all US cancer cases, including 78% of kidney cancers, 70% of bladder cancers, and 58% of prostate cancers.^28,29,30^

We excluded the 3 months before and after Medicaid expansion for expansion states and October 2013 through March 2014 for nonexpansion states to create a phase-in or wash-out period.^21^ We identified our sample by selecting primary site codes for kidney (C64), bladder (C670-C676, C678, or C679), and adenocarcinoma of the prostate (C619, histology code 8140) according to the International Classification of Disease for Oncology, Third edition,^31^ topography codes. For treatment-related outcomes, additional inclusion and exclusion criteria are detailed in eTable 1 in the Supplement. For treatment outcomes, patients diagnosed in the second half of 2016 were excluded for possible reporting lag.

### Outcomes and Covariates

Our outcomes were insurance status at the time of diagnosis (uninsured, Medicaid, private, or other), proportion of early-stage diagnosis (American Joint Committee on Cancer stage 1 for kidney cancer, American Joint Committee on Cancer stage 0-1 for bladder cancer, and National Comprehensive Cancer Network very low– or low-risk groups for prostate cancer), and a selection of cancer- and stage-specific treatment outcomes. Receipt of the first course of treatment, such as surgery, radiation, hormone therapy, and chemotherapy, including active surveillance or watchful waiting for prostate cancer, is reported in the NCDB.^32^

Demographic variables captured and categorized in the NCDB were age group at diagnosis (18-44, 45-54, or 55-64 years), sex (male or female), race/ethnicity (non-Hispanic White, non-Hispanic Black, non-Hispanic other, Hispanic, or unknown), zip code–level median income (<139% FPL, 139%-400% FPL, or >400% FPL), metropolitan statistical area (metropolitan, urban, rural, or unknown), Charlson-Deyo comorbidity score (0, 1, or ≥2), and facility case volume (disease specific and by quartile). The NCDB data are collected by electronic medical record review by trained abstractors. Race/ethnicity reflects is recorded in the patient’s medical record; however each participating institution may document race/ethnicity in the medical record by different means, and these means are not recorded by the NCDB.

### Statistical Analysis

We used χ^2^ tests to compare overall distribution of demographic variables between patients residing in expansion vs nonexpansion states. As a standard statistical approach for evaluating the association of health policy changes in quasi-experimental studies, difference-in-difference method was employed, which involves generating a linear probability regression for each outcome that contains binary variables indicating before or after and exposure or control, as well as an interaction variable.^33,34^ This interaction term describes the percentage point change associated with the exposure from before the exposure to after, while controlling for contemporaneous before to after changes in the control group. Our case group included patients in states that expanded Medicaid, and the control group included patients in states that did not expand Medicaid. The before and after periods were usually defined as 2010 to 2013 for pre-ACA Medicaid expansion and 2014 to 2016 for post-ACA Medicaid expansion. However, states that expanded Medicaid after January 2014 (ie, Michigan expanded Medicaid on April 1, 2014; New Hampshire, August 15, 2014; Pennsylvania, January 1, 2015; Indiana, February 1, 2015; Alaska, September 1, 2015; Montana, January 1, 2016; and Louisiana, July 1, 2016), were defined based on the actual expansion date. Absolute percentages of each of our outcomes were observed graphically over the entire study period, and the difference-in-difference parallel trends assumption was evaluated using 2013 as a placebo year of policy change for patients diagnosed before 2014 (eTable 2 in the Supplement).

We generated crude and adjusted difference-in-difference models controlling for age, sex, race/ethnicity, zip code–level income, and metropolitan statistical area status. We accounted for secular trends by including a continuous form of diagnosis year in the model, and accounted for clustering at the state level by using random effects modeling,^35^ as used in previous studies on Medicaid expansion and health care outcomes.^36,37^ The model equation is: *Y_ist_* = β_1_*expansion_s_* + β_2_*post_t_* + β_3_*expansion_s_* × *post_t_* + Σγ*_k_X_ik_* + δ_s_ + η*_t_* + ε*_ist_*, in which *i* indicates the individual patient; *s*, the state; and *t*, the year. The expansion and post variables indicate yes/no Medicaid expansion status and post-ACA expansion status. *X_ik_* indicates the *k* characteristic covariate controlled; *δ_s_*, random effects for each state; and *η_t_*, the linear time trend. *β_3_* in the regression specification is the difference-in-difference estimator for changes in outcome *Y* associated with Medicaid expansion after implementation of the ACA.

Charlson-Deyo comorbidity score and facility case volume were added to multivariable difference-in-difference models for treatment outcomes. Missing values were treated as a separate unknown category in the models. In addition to overall sample, we also conducted subset analyses stratifying by cancer type and limiting to patients living in low-income areas. To assess the robustness of difference-in-difference estimates to unmeasured confounders, we calculated the *E* values which represent the minimum strength of association that would be required between an unmeasured confounder and both state’s Medicaid expansion status and changes in disease outcomes to overcome the statistically significant outcome observed.^38^

All *P* values were 2-sided and deemed statistically significant at α = .05. All statistical analyses were conducted using SAS statistical software version 9.4 (SAS Institute). Data were analyzed from January 2020 to March 2021.

## Results

A total of 340 552 new diagnoses genitourinary cancers were identified in the NCDB in patients aged 18 to 64 years between 2011 to 2016, including 94 033 patients (27.6%) with kidney cancer, 25 770 patients (7.6%) with bladder cancer, and 220 749 patients (64.8%) with prostate cancer. Among these, 210 570 patients (61.8%) were in expansion states, and 129 982 patients (38.2%) were in nonexpansion states. Black and low-income patients were disproportionately represented in nonexpansion states (Table 1).

**Table 1.** Characteristics of Patients Newly Diagnosed With Genitourinary Malignant Neoplasms in the National Cancer Database from 2011 to 2016

Variable	No. (%)^a^				
	Total (n = 340 552)	Expansion states (n = 210 570)		Nonexpansion states (n = 129 982)	
		Pre-ACA	Post-ACA	Pre-ACA	Post-ACA
Primary neoplasm site					
Kidney	94 033 (27.6)	29 835 (25)	26 837 (29.3)	18 049 (26.5)	19 312 (31.2)
Bladder	25 770 (7.6)	9043 (7.6)	7408 (8.1)	4686 (6.9)	4633 (7.5)
Prostate	220 749 (64.8)	80 247 (67.4)	57 200 (62.6)	45 432 (66.6)	37 870 (61.3)
Diagnosis year					
2011	70 574 (20.7)	43 681 (36.7)	0	26 893 (39.5)	0
2012	61 995 (18.2)	38 319 (32.2)	0	23 676 (34.7)	0
2013	47 985 (14.1)	30 387 (25.5)	0	17 598 (25.8)	0
2014	43951 (12.9)	5096 (4.3)	22 024 (24.1)	0	16 831 (27.2)
2015	58 855 (17.3)	1372 (1.2)	34 246 (37.4)	0	23 237 (37.6)
2016	57 192 (16.8)	270 (0.2)	35 175 (38.5)	0	21 747 (35.2)
Age, y					
18-44	19 833 (5.8)	6372 (5.3)	5354 (5.9)	4084 (6)	4023 (6.5)
45-54	81 994 (24.1)	29 274 (24.6)	20 715 (22.7)	17 336 (25.4)	14 669 (23.7)
55-64	238 725 (70.1)	83 479 (70.1)	65 376 (71.5)	46 747 (68.6)	43 123 (69.8)
Race/ethnicity					
Non-Hispanic White	246 008 (72.2)	89 283 (74.9)	66 912 (73.2)	47 832 (70.2)	41 981 (67.9)
Non-Hispanic Black	59 751 (17.5)	17 766 (14.9)	13 142 (14.4)	14 718 (21.6)	14 125 (22.9)
Hispanic	21 205 (6.2)	6586 (5.5)	6550 (7.2)	4042 (5.9)	4027 (6.5)
Non-Hispanic other	9843 (2.9)	3862 (3.2)	3604 (3.9)	1160 (1.7)	1217 (2)
Unknown	3745 (1.1)	1628 (1.4)	1237 (1.4)	415 (0.6)	465 (0.8)
Sex					
Men	301 118 (88.4)	106 540 (89.4)	80 558 (88.1)	60 267 (88.4)	53 753 (87)
Women	39 434 (11.6)	12 585 (10.6)	10 887 (11.9)	7900 (11.6)	8062 (13)
Comorbidity score					
0	274 720 (80.7)	97 388 (81.8)	73 932 (80.8)	54 424 (79.8)	48 976 (79.2)
1	50 688 (14.9)	17 459 (14.7)	12 782 (14)	11 013 (16.2)	9434 (15.3)
≥2	15 144 (4.4)	4278 (3.6)	4731 (5.2)	2730 (4)	3405 (5.5)
Income, FPL					
Low (<139%)	25 915 (7.6)	7889 (6.6)	5566 (6.1)	6608 (9.7)	5852 (9.5)
Middle (139%-400%)	281 535 (82.7)	97 186 (81.6)	74 465 (81.4)	57 541 (84.4)	52 343 (84.7)
High (>400%)	32 315 (9.5)	13 703 (11.5)	11 217 (12.3)	3870 (5.7)	3525 (5.7)
Unknown	787 (0.2)	347 (0.3)	197 (0.2)	148 (0.2)	95 (0.2)
Residence					
Metropolitian	279 050 (81.9)	99 930 (83.9)	76 994 (84.2)	53 527 (78.5)	48 599 (78.6)
Urban	47 006 (13.8)	14 622 (12.3)	10 898 (11.9)	11 341 (16.6)	10 145 (16.4)
Rural	5987 (1.8)	1502 (1.3)	1147 (1.3)	1773 (2.6)	1565 (2.5)
Unknown	8509 (2.5)	3071 (2.6)	2406 (2.6)	1526 (2.2)	1506 (2.4)
Facility type					
Community	21 197 (6.2)	7225 (6.1)	5797 (6.3)	4016 (5.9)	4159 (6.7)
Comprehensive community	121 490 (35.7)	37 801 (31.7)	29 059 (31.8)	28 636 (42)	25 994 (42.1)
Teaching or research	86 047 (25.3)	33 797 (28.4)	25 722 (28.1)	13 317 (19.5)	13 211 (21.4)
NCI	60 491 (17.8)	22 271 (18.7)	19 139 (20.9)	9853 (14.5)	9228 (14.9)
Other^b^	51 327 (15.1)	18 031 (15.1)	11 728 (12.8)	12 345 (18.1)	9223 (14.9)
Facility volume^c^					
Very Low	11 311 (3.3)	4142 (3.5)	3234 (3.5)	2084 (3.1)	1851 (3)
Low	31 474 (9.2)	11 562 (9.7)	9270 (10.1)	5111 (7.5)	5531 (8.9)
Medium	70 925 (20.8)	24 285 (20.4)	19 461 (21.3)	14 048 (20.6)	13 131 (21.2)
High	22 6842 (66.6)	79 136 (66.4)	59 480 (65)	46 924 (68.8)	41 302 (66.8)

Abbreviations: ACA, Patient Protection and Affordable Care Act; FPL, federal poverty line; NCI, National Cancer Institute.

^a^ Patients diagnosed 3 months before or 3 months after Medicaid expansion in expansion states and patients diagnosed in October 2013 to March 2014 in nonexpansion states were excluded. Missing or unknown values not shown in table.

^b^ Other facility type included Integrated Network Cancer Program, Hospital Associate Cancer Program, Pediatric Cancer Program, Free Standing Cancer Center Program.

^c^ Facility volumes were calculated as the number of patients treated in the facility in a year and categorized based on quartiles: very low indicates 1 to 3 kidney cancer cases, 1 bladder cancer case, or 1 to 6 prostate cancer cases; low, 4 to 7 kidney cancer cases, 2 bladder cancer cases, or 7 to 16 prostate cancer cases; medium, 8 to 16 kidney cancer cases, 3 to 4 bladder cancer cases, or 7 to 16 prostate cancers; high, 17 or more kidney cancer cases, 5 or more bladder cancer cases, or 38 or more prostate cancer cases.

### Changes in Insurance Status

Medicaid expansion was associated with a net increase of 4.5 (95% CI, 4.2 to 4.9) percentage points in the proportion of patients with Medicaid insurance, a net decrease of 3.1 (95% CI, −3.6 to −2.5) percentage points in patients with private insurance, and a net decrease of 1.1 (95% CI, −1.4 to −0.8) percentage points in patients who were uninsured. These net changes were even larger in the low-income population, with an increase of 9.8 (95% CI, 8.0 to 11.6) percentage points in patients enrolled in Medicaid, a decrease of 3.6 (95% CI, −6.1 to −1.2) percentages in patients with private insurance, and a decrease of 4.4 (95% CI, −5.7 to −3.0) in patients who were uninsured (eTable 3 in the Supplement). In expansion states, there was a decrease in the proportion of patients who were uninsured (absolute percentage change [APC], −2.3 [95% CI, −2.5 to −2.2] percentage points), driven mainly by a proportional increase in Medicaid insurance (APC, 5.0 [95% CI, 4.8 to 5.3] percentage points). By contrast, the decrease in patients who were uninsured in nonexpansion states (APC, −1.2 [95% CI, −1.4 to −0.9] percentage points) was smaller and wholly associated with an increase in privately insured patients (APC, 0.5 [95% CI, 0.0 to 1.0] percentage points) rather than Medicaid. The eFigure and eTable 4 in the Supplement show the data biannually to better describe these trends.

### Changes in Cancer Stage

Medicaid expansion was associated with a net increase of 1.4 (95% CI, 0.1 to 2.6) percentage points in the proportion of kidney cancers diagnosed at stage 1 (Table 2). For the low-income group, the net increase was 4.6 (95% CI, 0.3 to 9.0) percentage points.

**Table 2.** Cancer Diagnosis at an Early Stage from the Pre-ACA and Post-ACA Periods by Medicaid Expansion Status

Population	Patients, No.	Early stage at diagnosis, No. (%)						Model			
		Medicaid expansion states			Medicaid nonexpansion states			Crude		Adjusted^a^	
		Pre-ACA	Post-ACA	APC (95% CI)	Pre-ACA	Post-ACA	APC (95% CI)	Difference-in-difference, % (95% CI)	*P* value	Difference-in-difference, % (95% CI)	*P* value
All **incomes**											
Kidney cancer stage 1	94 033	18 119 (60.7)	16 607 (61.9)	1.2 (0.3 to 2)^a^	11 037 (61.2)	11 750 (60.8)	−0.3 (−1.3 to 0.7)	1.5 (0.2 to 2.7)^a^	.03	1.4 (0.1 to 2.6)^a^	.04
Bladder cancer stage 0-1	25 770	4078 (45.1)	3353 (45.3)	0.2 (−1.4 to 1.7)	1943 (41.5)	1901 (41.0)	−0.4 (−2.4 to 1.6)	0.6 (−1.9 to 3.1)	.64	0 (−2.5 to 2.5)	.99
Prostate cancer low risk^b^	220 749	25 259 (31.5)	14 724 (25.7)	−5.7 (−6.2 to −5.3)^a^	14 096 (31.0)	9526 (25.2)	−5.9 (−6.5 to −5.3)^a^	0.1 (−0.6 to 0.9)	.73	−0.2 (−0.9 to 0.6)	.69
**Low-income**											
Kidney cancer stage 1	7681	1333 (60.8)	1154 (64.6)	3.9 (0.8 to 6.9)^a^	1113 (61.2)	1132 (60.1)	−1.0 (−4.2 to 2.1)	4.9 (0.5 to 9.3)^a^	.03	4.6 (0.3 to 9)^a^	.04
Bladder cancer stage 0-1	1883	214 (37.2)	164 (38.4)	1.2 (−4.9 to 7.3)	167 (36.9)	143 (33.3)	−3.6 (−9.9 to 2.7)	4.8 (−3.9 to 13.6)	.28	3.0 (−5.7 to 11.7)	.50
Prostate cancer low risk^b^	16 351	1269 (24.8)	719 (21.4)	−3.3 (−5.2 to −1.5)^a^	1190 (27.4)	752 (21.2)	−6.2 (−8.1 to −4.3)^a^	2.9 (0.2 to 5.5)^a^	.03	3.0 (0.3 to 5.7)^a^	.03

Abbreviation: ACA, Patient Protection and Affordable Care Act; APC, absolute percentage change.

^a^ This 95% CI does not overlap with 0; *P* < .05.

^b^ Low-risk group defined according to National Comprehensive Cancer Network guidelines for very low– or low- risk strata (Gleason score ≤6; clinical T ≤T2a; prostate-specific antigen <10).

**Figure 1.**
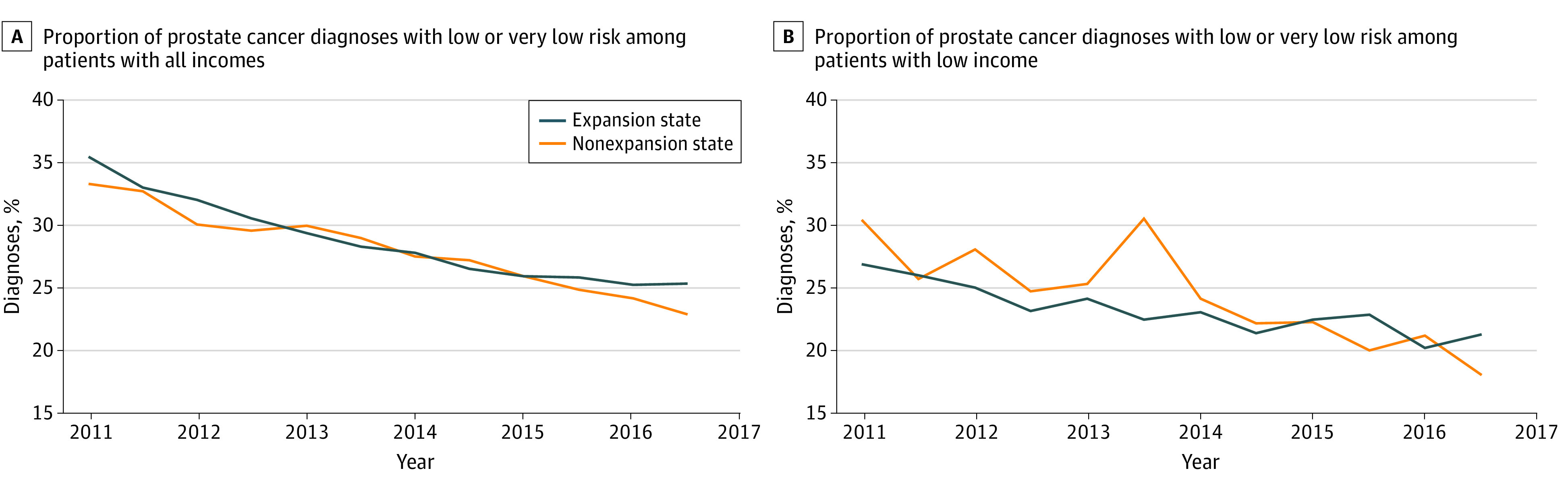
Trend of Low-Risk Diagnosis for Prostate Cancer in All-Income and Low-Income Populations

In prostate cancer, there was a steady decline in the proportion of diagnoses made at early stage in expansion (APC, −5.7 [95% CI, −6.2 to −5.3] percentage points) and nonexpansion (APC, −5.9 [95% CI, −6.5 to −5.3] percentage points) states (Table 2). In 2014, the decline did not change course in nonexpansion states but plateaued slightly in expansion states (Figure 1; eTable 5 in the Supplement), with a smaller magnitude decrease for expansion states. This is particularly true in the low-income population, in which the APC was −6.2 (95% CI, −8.1 to −4.3) percentage points for nonexpansion states and −3.3 (95% CI, −5.2 to −1.5) percentage points for expansion states. In the adjusted model, the difference-in-difference estimate was a net increase of 3.0 (95% CI, 0.3 to 5.7) percentage points in early-stage diagnoses associated with expansion.

### Changes in Treatment

Table 3 shows the results from the difference-in-difference analyses to detect associations between Medicaid expansion and changes in treatment. For kidney cancer, APCs show the proportion of stage 0 to 3 cancers receiving resection decreased, coupled with increase in use of biopsy and active surveillance in expansion and nonexpansion states. The percentage of patients receiving biopsy had the largest magnitude of increase, with an increase of 6.5 (95% CI, 4.9 to 8.1) percentage points in expansion states and 4.8 (95% CI, 3.1 to 6.5) percentage points in nonexpansion states. In adjusted models, the difference-in-difference estimator was a net increase of 1.5 (95% CI, −0.8 to 3.8) percentage points in expansion states compared with nonexpansion states, but this result was no longer statistically significant.

**Table 3.** Changes in Treatment from the Pre-ACA and Post-ACA Periods by Medicaid Expansion Status

Cancer stage	Treatment Type	Patients, No.	Receiving treatment, No. (%)						Model			
			Medicaid expansion states			Medicaid non-expansion states			Crude		Adjusted^a^	
			Pre-ACA	Post-ACA	APC (95% CI)	Pre-ACA	Post-ACA	APC (95% CI)	Difference-in-difference, % (95% CI)	*P* value	Difference-in-difference, % (95% CI)	*P* value
**All incomes**												
Kidney cancer												
Stage 0-3	Resection	70 173	22 471 (98.0)	17 513 (97.7)	−0.3 (−0.6 to 0)^b^	15 922 (97.7)	12 645 (97.0)	−0.6 (−1.0 to −0.3)^b^	0.3 (−0.2 to 0.8)	.21	0.3 (−0.1 to 0.8)	.16
Stage T1aN0M0	Biopsy	13 862	492 (10.6)	508 (17.1)	6.5 (4.9 to 8.1)^b^	399 (10.6)	385 (15.4)	4.8 (3.1 to 6.5)^b^	1.7 (−0.7 to 4)	.16	1.5 (−0.8 to 3.8)	.19
Stage T1aN0M0	AS	13 862	37 (0.8)	46 (1.5)	0.7 (0.2 to 1.3)^b^	16 (0.4)	37 (1.5)	1.1 (0.5 to 1.6)^b^	−0.3 (−1 to 0.4)	.41	−0.3 (−0.9 to 0.4)	.45
Bladder cancer												
Stages 0-1	Resection	9996	3740 (98.3)	2639 (98.8)	0.6 (0 to 1.2)	1951 (97.7)	1500 (98.5)	0.8 (−0.1 to 1.7)	−0.2 (−1.3 to 0.8)	.69	−0.2 (−1.2 to 0.9)	.74
Stages 2-3	RC or trimodal therapy	6439	1224 (52.8)	859 (54.4)	1.6 (−1.5 to 4.8)	731 (50.9)	596 (54.0)	3.1 (−0.8 to 7)	−1.4 (−6.5 to 3.6)	.58	−1.6 (−6.6 to 3.3)	.52
Stage 2-3	RC and NAC	3104	329 (29.7)	287 (36.1)	6.4 (2.1 to 10.7)^b^	176 (26.9)	208 (38.2)	11.4 (6.1 to 16.7)^b^	−5 (−11.8 to 1.8)	.15	−5.9 (−12.7 to 0.9)	.09
Prostate cancer												
Low-risk^c^	AS	59 415	2720 (11.3)	2971 (24.7)	13.5 (12.6 to 14.3)^b^	1165 (7.6)	1311 (16.3)	8.6 (7.7 to 9.6)^b^	4.8 (3.5 to 6.1)^b^	<.001	4.1 (2.9 to 5.3)^b^	<.001
High-risk^d^	Prostatectomy or radiation	84 665	17 463 (95.4)	20 656 (92.8)	−2.6 (−3 to −2.1)^b^	17 622 (93.9)	13 670 (92.0)	−2.0 (−2.5 to −1.4)^b^	−0.6 (−1.3 to 0.1)	.10	−0.5 (−1.2 to 0.2)	.13
**Low-income**												
Kidney cancer												
Stage 0-3	Resection	5676	1502 (97.1)	1166 (96.5)	−0.6 (−1.9 to 0.8)	1609 (96.8)	1196 (95.0)	−1.8 (−3.3 to −0.3)^b^	1.2 (−0.7 to 3.2)	.23	1.2 (−0.8 to 3.1)	.23
Stage T1aN0M0	Biopsy	1192	35 (11.2)	42 (17.9)	6.7 (0.7 to 12.8)^b^	43 (10.9)	37 (14.7)	3.8 (−1.6 to 9.1)	3.0 (−5.1 to 11)	.47	3.0 (−4.9 to 10.9)	.46
Stage T1aN0M0	AS	1192	<10 (0.3)	<10 (3.4)	3.1 (0.7 to 5.5)^b^	<10 (0.8)	<10 (2.8)	2.0 (−0.2 to 4.2)	1.1 (−2.2 to 4.3)	.51	1.0 (−1.9 to 3.9)	.50
Bladder cancer												
Stages 0-1	Resection	603	177 (96.2)	127 (97.7)	2.5 (−1.5 to 6.6)	167 (97.1)	113 (98.3)	1.2 (−2.3 to 4.6)	1.4 (−3.9 to 6.7)	.62	0.7 (−5.0 to 6.4)	.80
Stages 2-3	RC or trimodal therapy	494	68 (44.4)	41 (44.1)	−0.4 (−13.2 to 12.4)	66 (46.8)	51 (47.7)	0.9 (−11.7 to 13.4)	−1.2 (−19.1 to 16.7)	.89	−4.8 (−22.8 to 13.2)	.60
Stage 2-3	RC and NAC	198	21 (35.0)	14 (38.9)	3.9 (−16.1 to 23.9)	15 (25.0)	16 (38.1)	13.1 (−5.2 to 31.4)	−9.2 (−36.3 to 17.9)	.51	−11.1 (−40.0 to 17.8)	.45
Prostate cancer												
Low-risk^c^	AS	3698	128 (11.6)	147 (24.8)	13.2 (9.2 to 17.2)^b^	102 (7.5)	98 (15.2)	7.6 (4.5 to 10.7)^b^	5.6 (0.5 to 10.6)^b^	.03	4.5 (0 to 9)^b^	.05
High-risk^d^	Prostatectomy or radiation	6361	1576 (90.4)	1150 (88.4)	−2.0 (−4.2 to 0.2)	1798 (91.7)	1220 (89.9)	−1.8 (−3.8 to 0.2)	−0.2 (−3.2 to 2.8)	.90	−0.3 (−3.3 to 2.7)	.85

Abbreviations: ACA, Patient Protection and Affordable Care Act; APC, absolute percent change; AS, active surveillance; NAC, neoadjuvant chemotherapy; RC, radical cystectomy.

^a^ Models adjusted for age, race/ethnicity, sex, zip code–level income, region, metropolitan statistical area, number of comorbidities, facility volume, secular year, and state.

^b^ This 95% CI does not overlap with 0; *P* ≤ .05.

^c^ Low-risk group defined according to National Comprehensive Cancer Network guidelines for very low– or low-risk strata (Gleason score ≤6; clinical T ≤T2a; prostate-specific antigen <10).

^d^ High-risk group defined according to National Comprehensive Cancer Network guidelines for intermediate- or high-risk strata (Gleason score >6; clinical T >T2a; prostate-specific antigen ≥10).

In bladder cancer, the proportions of early-stage cancers receiving resection were high and unassociated with Medicaid expansion. By contrast, the proportion of patients receiving radical cystectomy for muscle invasive bladder cancer was low, and the proportion of patients who then also received the indicated neoadjuvant chemotherapy was even lower (Table 3). While the proportion of patients receiving neoadjuvant chemotherapy increased in expansion (APC, 6.4 [95% CI, 2.1 to 10.7] percentage points) and nonexpansion states (APC, 11.4 [95% CI, 6.1 to 16.7]), there was no statistically significant net change associated with Medicaid expansion.

**Figure 2.**
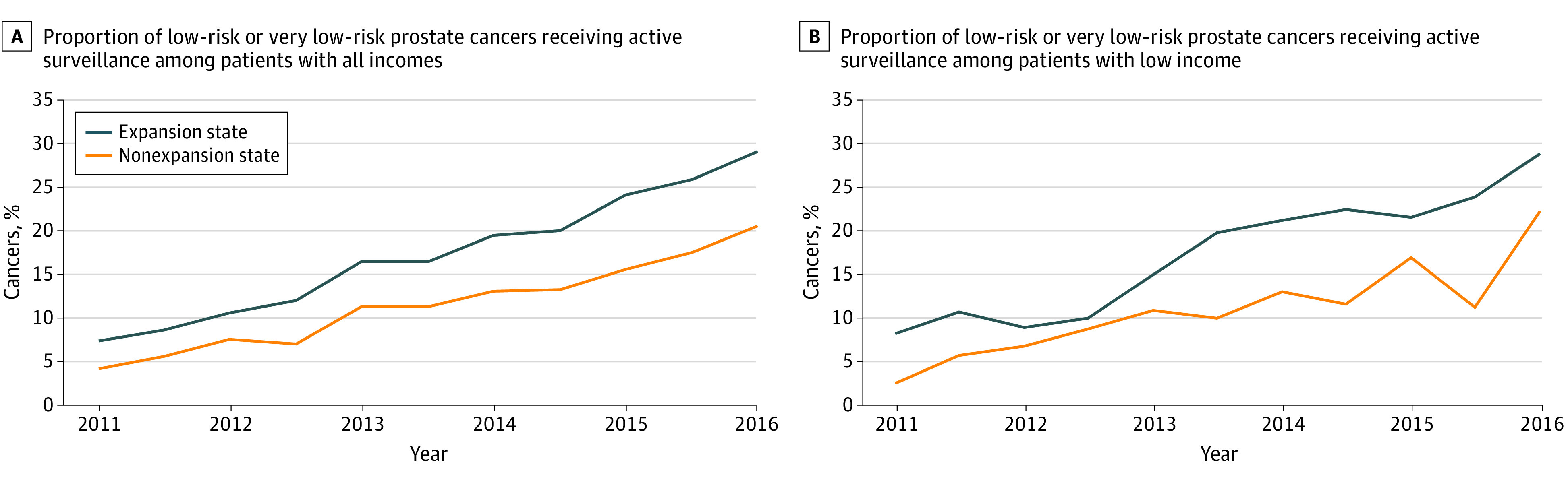
Trend of Patients With Low-Risk Prostate Cancer Patients Receiving Active Surveillance for All-Income and Low-Income Populations

For prostate cancer, the percentages of treatment for National Comprehensive Cancer Network intermediate- and high-risk localized disease decreased from before to after expansion time periods in expansion (APC, −2.6 [95% CI, −3.0 to −2.1] percentage points) and nonexpansion states (APC, −2.0 [95% CI, −2.5 to −1.4] percentage points), and there was no net difference associated with Medicaid expansion. The proportion of patients with low-risk disease who underwent active surveillance increased throughout the study in expansion (APC, 13.5 [95% CI, 12.6 to 14.3] percentage points) and nonexpansion (APC, 8.6 [95% CI, 7.7 to 9.6] percentage points) states (Figure 2; eTable 6 in the Supplement). In the adjusted model, there was a net increase of 4.1 (95% CI, 2.9 to 5.3) percentage points associated with Medicaid expansion across incomes and a net increase of 4.5 (95% CI, 0 to 9.0) percentage points among patients in low-income areas.

*E* values to estimate the robustness of the observed associations to unmeasured confounding suggested extensive unmeasured confounding would be required to eliminate observed associations between Medicaid expansion and changes in outcomes (eTable 7 in the Supplement). For example, the observed association of Medicaid expansion and increased diagnosis at an early stage of kidney cancer could be explained by an unmeasured confounder that was associated with Medicaid expansion and changes in stage at diagnosis by a risk ratio of 3.4 each, above and beyond the measured confounds, but weaker confounding could not do so (eTable 7 in the Supplement).

## Discussion

In this case-control study, we evaluated associations between Medicaid expansion and changes in insurance, stage at diagnosis, and treatment in patients with newly diagnosed bladder, kidney, or prostate cancers. Our findings are consistent with earlier studies describing Medicaid’s association with reductions in uninsured status and shifts toward earlier-stage disease at diagnosis for non-Hodgkin lymphoma and pancreatic, liver, and thyroid cancer.^21,22^ To our knowledge, our study is the first to associate Medicaid expansion with a stage shift for kidney and prostate cancer and also with an increase in active surveillance of low-risk prostate cancer.

One of the most important takeaways from our study is the greater magnitude of all detected changes in the low-income subanalysis compared with the entire population. Genitourinary malignant neoplasms display varying degrees of racial/ethnic, sex, and socioeconomic disparities not only in cancer survival but throughout the diagnosis and treatment process. In some genitourinary cancers, insurance has been shown to act as an association modifier for these variables,^16,39,40^ indicating it may be a powerful tool to reduce disparity in cancer care and, ultimately, outcomes. The decrease in uninsured status associated with Medicaid expansion in our study was 1.1 percentage points across all incomes, but 4.4 percentage points in the low-income group. This trend is consistent with other studies that have shown that Medicaid expansion was associated with reduced socioeconomic disparity in insurance rates.^20,21,22^ Importantly, our findings suggest that the downstream stage and treatment outcomes were also magnified in the low-income population. The fact that changes in the low-income population are associated with trends toward earlier diagnosis and receipt of indicated treatment suggests that expansion of insurance may be a valid mechanism to help reduce cancer disparity.

The association between gaining insurance and improved cancer outcomes is likely multifactorial and variable between different cancer types. For prostate cancer, an association between gaining insurance and undergoing prostate-specific antigen (PSA) screening could explain the association our study identified between insurance and early-stage diagnosis. Complicating this explanation is the fact that recent studies have reported that the practice of PSA screening has been decreasing over the past decade,^41,42,43^ and that this decrease was associated with decreasing incidence both overall^42,43,44,45^ and specifically incidence of early-stage cancers.^43^ Our findings agree with this trend by showing that low-risk prostate cancer has decreased in both Medicaid expansion and nonexpansion states; however, our data also suggest that the rate of decrease was slower in Medicaid expansion states, yielding a net increase in early-stage disease associated with Medicaid expansion. Furthermore, despite the US Preventive Services Task Force’s 2012 recommendation against PSA screening and mixed results associated with screening in other nongenitourinary cancers associated with the ACA,^46^ a 2018 study by Sammon et al^47^ showed that between 2012 and 2014, there was an increase in self-reported rates of PSA screening associated with early expansion of Medicaid. In general, studies have shown that insurance status^48,49^ and physician access^50^ increase rates of PSA screening. Thus, while our study does not attempt to identify PSA screening as a factor, it does offer a potential explanation for how Medicaid expansion is associated with moderating an ongoing decrease in early-stage prostate cancer detection.

Critically, our data show that this net shift to earlier stage prostate cancer diagnosis was accompanied by an increase in active surveillance associated with Medicaid expansion. The dual existence of early detection via PSA screening and active surveillance is essential in building a strong approach to prostate cancer care. Modeling studies suggest that 23% to 42% of all prostate cancers in the US detected in screening examinations were overtreated.^51^ PSA screening has been shown to be associated with a 40% reduction in prostate cancer death,^52^ but PSA screening will continue to be controversial without a reduction in overtreatment. It has been demonstrated that active surveillance is a viable and recommended option for patients with low-risk and very low–risk prostate cancer to avoid overtreatment,^53,54^ and active surveillance is now considered the preferred option by multiple professional organizations.^55^ There can be significant cost savings for patients undergoing active surveillance compared with up-front radical prostatectomy, potentially representing a 43% to 79% cost savings.^56^ Studies have reported that campaigns to increase the use of active surveillance have been largely successful,^57,58^ which is consistent with our detected absolute increases of 13.5% in expansion states and 8.6% in nonexpansions states. However, many studies have found that active surveillance is overall still underused, and its utilization is variable among different practices and regions throughout the US.^59,60^ To our knowledge, our study is the first to show an increase in use of active surveillance associated specifically with Medicaid expansion.

In contrast to the shift in stage at detection we observed in prostate cancer, the association between Medicaid expansion and the observed shift in stage at detection for kidney cancer cannot be explained by an increase in screening. There is no effective screening test for kidney cancer. However, incidental diagnoses make up a significant and increasing portion of kidney cancer diagnoses, and this may offer an explanation for the association between Medicaid expansion and earlier-stage diagnosis of kidney cancer. Researchers have postulated that increased use of health care services, particularly chest and abdominal imaging, was associated with the large increase in incidence as well as a shift toward earlier-stage detection of kidney cancer observed in the 1990s and early 2000s.^61,62,63,64,65^ In the years surrounding Medicaid expansion, the incidence of kidney cancer in the US was relatively unchanged. However, studies have shown that the Medicaid expansion was associated with increased preventive care visits^66^ and increased outpatient visits.^67^ Thus, there is a similar potential explanation wherein the increased access to care and resources afforded by Medicaid expansion may lead to increased incidental diagnosis at early stages when kidney cancer is still asymptomatic. Unlike in prostate cancer, our data do not detect a corresponding shift toward active surveillance, although they do indicate that active surveillance for kidney cancer increased in expansion states by 0.7% and in nonexpansion states by 1.1%.

### Limitations

This study has some limitations. One potential limitation of this study is the geographic variability in the proportion of cancer cases captured in the NCDB.^66^ Furthermore, Commission on Cancer–accredited hospitals are more likely to be larger, academic, urban facilities that offer more cancer-related services, such as screening, chemotherapy, and radiation.^68^ However, previous analyses, such as a 2018 study by Eguia et al,^27^ have reported that most demographic and clinical characteristics are remarkably similar between the NCDB and the population-based Surveillance, Epidemiology, and End Results database. Another limitation is that our low-income population was only able to be defined with zip code–level median income owing to lack of individual income information. Additionally, while our study represents the most recently available data, several additional states have expanded Medicaid coverage since 2016, and these ongoing expansions highlight the need for continued research to include these states as well as to assess outcomes that may require more than 3 years to reflect outcomes associated with Medicaid expansion.

## Conclusions

This case-control study found that Medicaid expansion was associated not only with reductions in uninsured status, but also with shifts toward earlier stages at diagnosis among kidney and prostate cancers and higher rates of active surveillance among patients with low-risk prostate cancer. All these outcomes were larger in magnitude in patients residing in low-income areas. This finding has potential implications in that it shows expanded insurance may have positive impact on practice patterns in cancer management, particularly in reducing inequity.

## Notes

Supplement. **eTable 1.** Additional Inclusion and Exclusion Criteria and Treatment Definitions **eTable 2.** Evaluation of Parallel Trend Assumption for Difference-in-Differences Analysis Using 2013 as a Placebo Year of Policy Change, NCDB 2011-2013 **eTable 3.** Changes in Health Insurance Coverage Between the Pre-ACA and Post-ACA Periods by Medicaid Expansion Status **eTable 4.** Percentages of Health Insurance Type by Medicaid Expansion Status for All-Income and Low-Income Populations by 6-Month Increments **eTable 5.** Percentages of Low Risk Diagnoses for Prostate Cancer in All-Income and Low-Income Populations by 6-Month Increments **eTable 6.** Percentage of Patients With Low-Risk Prostate Cancer Receiving Active Surveillance for All-Income and Low-Income Populations by 6-Month Increments **eTable 7.** Assessment of Robustness of Statistically Significant Associations Observed to Unmeasured Confounding **eFigure.** Changes in Percentages of Health Insurance Type by Medicaid Expansion Status for All-Income and Low-Income Populations
