# Supplementary material for: Associations of Medicaid Expansion With Insurance Coverage, Stage at Diagnosis, and Treatment Among Patients With Genitourinary Malignant Neoplasms
Source: JAMA Netw Open. 2021 May 19;4(5):e217051. doi: 10.1001/jamanetworkopen.2021.7051 (PMC8134994; doi:10.1001/jamanetworkopen.2021.7051)
Supplement: Supplement. — eTable 1. Additional Inclusion and Exclusion Criteria and Treatment Definitions eTable 2. Evaluation of Parallel Trend Assumption for Difference-in-Differences Analysis Using 2013 as a Placebo Year of Policy Change, NCDB 2011-2013 eTable 3. Changes in Health Insurance Coverage Between the Pre-ACA and Post-ACA Periods by Medicaid Expansion Status eTable 4. Percentages of Health Insurance Type by Medicaid Expansion Status for All-Income and Low-Income Populations by 6-Month Increments eTable 5. Percentages of Low Risk Diagnoses for Prostate Cancer in All-Income and Low-Income Populations by 6-Month Increments eTable 6. Percentage of Patients With Low-Risk Prostate Cancer Receiving Active Surveillance for All-Income and Low-Income Populations by 6-Month Increments eTable 7. Assessment of Robustness of Statistically Significant Associations Observed to Unmeasured Confounding eFigure. Changes in Percentages of Health Insurance Type by Medicaid Expansion Status for All-Income and Low-Income Populations [file jamanetwopen-e217051-s001.pdf]

Supplemental Online Content

Michel KF, Spaulding A, Jemal A, Yabroff KR, Lee DJ, Han X. Associations of Medicaid expansion with insurance coverage, stage at diagnosis, and treatment among patients with genitourinary malignant neoplasms. *JAMA Netw Open*. 2021;4(5):e217051. doi:10.1001/jamanetworkopen.2021.7051

eTable 1. Additional Inclusion and Exclusion Criteria and Treatment Definitions

eTable 2. Evaluation of Parallel Trend Assumption for Difference-in-Differences Analysis Using 2013 as a Placebo Year of Policy Change, NCDB 2011-2013

eTable 3. Changes in Health Insurance Coverage Between the Pre-ACA and Post-ACA Periods by Medicaid Expansion Status

eTable 4. Percentages of Health Insurance Type by Medicaid Expansion Status for All-Income and Low-Income Populations by 6-Month Increments

eTable 5. Percentages of Low Risk Diagnoses for Prostate Cancer in All-Income and Low-Income Populations by 6-Month Increments

eTable 6. Percentage of Patients With Low-Risk Prostate Cancer Receiving Active Surveillance for All-Income and Low-Income Populations by 6-Month Increments

eTable 7. Assessment of Robustness of Statistically Significant Associations Observed to Unmeasured Confounding

eFigure. Changes in Percentages of Health Insurance Type by Medicaid Expansion Status for All-Income and Low-Income Populations

This supplemental material has been provided by the authors to give readers additional information about their work.

© 2021 Michel KF et al. *JAMA Network Open.*

eTable 1. Additional Inclusion and Exclusion Criteria and Treatment Definitions

Population	Additional Inclusion/Exclusion Criteria	Treatment Outcome
Kidney Cancer		
All stages		Any treatment
Stage 0-3	Excluded if patients refused or ineligible for surgery	Resection (Any surgical code in the FORDS appendix: 10-15, 20-27, 30, 40, 50, 70, 80, 90)
Stage T1aN0M0		Biopsy (variable in NCDB)
Stage T1aN0M0		Active surveillance (variable in NCDB)
Bladder Cancer		
All Stages		Any treatment
Stages 0-1	Restricted to urothelial bladder cancer (histology codes: 8120, 8130, 8131, 8082, 8122, 8031, and 8020)<br><br>Excluded if patients refused or ineligible for surgery	Resection (Any surgical code in the FORDS appendix: 10-16, 20-27, 30, 50, 60-64, 70-74, 80, 90)
Stages 2-3	Restricted to urothelial bladder cancer (histology codes: 8120, 8130, 8131, 8082, 8122, 8031, and 8020)<br><br>Excluded if patients refused or ineligible for surgery	RC (FORDS appendix: 50, 60-64, 70-74) or trimodal therapy (FORDS appendix 20-25, 30 with any chemotherapy and any radiation)
Stage 2-3 who received radical cystectomy	Restricted to urothelial bladder cancer (histology codes: 8120, 8130, 8131, 8082, 8122, 8031, and 8020)<br><br>Excluded if patients refused or ineligible for chemotherapy	Neoadjuvant chemotherapy (any kind or amount, must have received prior to radical cystectomy, as coded above)
Prostate Cancer		
All stages		Any treatment
Low risk	Clinical T<=T2a<br>AND biopsy Gleason score <=6<br>AND PSA<10	Active surveillance (variable in NCDB)
High risk	Any clinical T>2a (including T2)<br>OR biopsy Gleason score>6<br>OR PSA >=10<br><br>AND M=0 (“localized”) and N=0	Prostatectomy (FORDS appendix 50 or 70) or radiation

APC= Absolute Percent Change. ppt=percentage points. DD=difference-in-difference. AS=active surveillance. RC=radical cystectomy

© 2021 Michel KF et al. *JAMA Network Open.*

eTable 2. Evaluation of Parallel Trend Assumption for Difference-in-Differences Analysis Using 2013 as a Placebo Year of Policy Change, NCDB 2011-2013

		Expansion States, ppt			Nonexpansion States, ppt			Crude		Adjusted*	
		2011-2013	2014-2016	APC (95% CI)	2011-2013	2014-2016	APC (95% CI)	DD, ppt	P Value	DD, ppt	P value
All income combined											
Kidney	Uninsured	5.6	5.6	0 (-0.6, 0.6)	9.8	10.6	0.8 (-0.2, 1.8)	-0.8 (-2, 0.4)	0.17	-0.5 (-1.6, 0.6)	0.35
	Medicaid	10.5	11.2	0.7 (-0.1, 1.5)	8.2	7.9	-0.3 (-1.1, 0.6)	1 (-0.2, 2.1)	0.11	1.1 (-0.1, 2.3)	0.06
	Private	70.3	68.6	-1.7 (-2.9, -0.5)	65.2	63.7	-1.5 (-3, 0.1)	-0.2 (-2.2, 1.7)	0.81	-0.6 (-2.5, 1.3)	0.51
	Stage 1	60.8	60.3	-0.5 (-1.8, 0.7)	61.4	60.4	-1 (-2.6, 0.6)	0.4 (-1.6, 2.5)	0.68	0.3 (-1.7, 2.4)	0.74
	Surgery among stage 0-3	98.3	97.4	-0.9 (-1.3, -0.5)	97.8	97.3	-0.5 (-1, 0.1)	-0.4 (-1.1, 0.3)	0.23	-0.4 (-1.1, 0.2)	0.21
	Biopsy among stage T1aN0M0	10.3	11.6	1.3 (-0.8, 3.3)	9.7	12.2	2.4 (0.1, 4.8)	-1.2 (-4.3, 1.9)	0.46	-1.1 (-4, 1.9)	0.49
	Active surveillance among stage T1aN0M0	0.8	0.9	0.1 (-0.5, 0.7)	0.3	0.7	0.4 (-0.2, 1)	-0.3 (-1.2, 0.5)	0.43	-0.4 (-1.2, 0.4)	0.35
Bladder	Uninsured	6.6	8.6	2 (0.7, 3.3)	14.1	14.1	0 (-2.2, 2.2)	2 (-0.6, 4.6)	0.12	1.6 (-0.7, 3.9)	0.16
	Medicaid	12.6	12.8	0.3 (-1.3, 1.8)	10.9	10.3	-0.6 (-2.5, 1.4)	0.8 (-1.7, 3.3)	0.53	1.1 (-1.4, 3.6)	0.40
	Private	66.6	64	-2.5 (-4.8, -0.3)	59.2	57.4	-1.8 (-4.9, 1.3)	-0.7 (-4.6, 3.1)	0.70	-0.6 (-4.3, 3.1)	0.75
	Stage 0-1	45.4	45.8	0.4 (-1.9, 2.8)	41.4	41.6	0.1 (-3, 3.3)	0.3 (-3.6, 4.2)	0.88	0.1 (-3.8, 4)	0.96
	Resection among stage 0-1	98	98.9	0.8 (0, 1.7)	97.6	98.2	0.6 (-0.8, 2)	0.3 (-1.4, 1.9)	0.76	0.3 (-1.4, 2)	0.72
	Radical cystectomy or trimodal therapy among stage 2-3	52.7	50.7	-2 (-6.6, 2.7)	50.2	51.7	1.5 (-4.2, 7.3)	-3.5 (-10.9, 3.9)	0.36	-4.4 (-11.7, 2.8)	0.23
	Neoadjuvant chemotherapy among stage 2-3 who received radical cystectomy	29.1	29.6	0.5 (-5.7, 6.8)	24.6	30.4	5.8 (-1.9, 13.6)	-5.3 (-15.2, 4.7)	0.30	-5.2 (-15.1, 4.6)	0.30
Prostate	Uninsured	3	2.8	-0.2 (-0.4, 0.1)	4.8	5.2	0.4 (0, 0.9)	-0.6 (-1.1, -0.1)	0.03	-0.6 (-1.1, -0.1)	0.02
	Medicaid	4.6	5.4	0.8 (0.4, 1.1)	3.2	3.6	0.4 (0, 0.7)	0.4 (-0.1, 0.9)	0.14	0.5 (-0.1, 1)	0.08
	Private	83.2	81.7	-1.5 (-2.2, -0.9)	79.5	78.2	-1.3 (-2.2, -0.4)	-0.2 (-1.3, 0.9)	0.69	-0.1 (-1.1, 0.9)	0.81
	Low risk	32.7	28.8	-3.9 (-4.7, -3.2)	31.5	29.6	-1.9 (-2.8, -0.9)	-2.1 (-3.3, -0.8)	<0.001	-1.9 (-3.1, -0.6)	<0.001

© 2021 Michel KF et al. *JAMA Network Open.*

	Active Surveillance among low risk	9.4	16.5	7.1 (6, 8.2)	5.9	11.5	5.5 (4.4, 6.6)	1.6 (0, 3.1)	0.05	1.4 (0, 2.8)	0.05
	Prostatectomy or radiation among high risk	95.7	94.7	-1 (-1.6, -0.5)	94.1	93.9	-0.1 (-0.9, 0.6)	-0.9 (-1.9, 0.1)	0.07	-0.9 (-1.8, 0.1)	0.06
Low-income											
Kidney	Uninsured	10.2	9.5	-0.7 (-3.5, 2.1)	14.2	14.7	0.5 (-3.1, 4.1)	-1.2 (-5.7, 3.4)	0.62	0.2 (-4.3, 4.6)	0.93
	Medicaid	20	22.3	2.3 (-1.6, 6.2)	15.1	14.9	-0.3 (-3.9, 3.4)	2.6 (-2.8, 7.9)	0.34	3.1 (-2.2, 8.3)	0.26
	Private	46.1	42.7	-3.5 (-8.2, 1.3)	47	45.6	-1.4 (-6.5, 3.6)	-2 (-8.9, 4.9)	0.57	-2.4 (-9.2, 4.5)	0.50
	Stage 1	60.8	59.6	-1.2 (-5.8, 3.5)	61.3	60.8	-0.5 (-5.4, 4.5)	-0.7 (-7.5, 6.1)	0.85	-1.2 (-8, 5.6)	0.73
	Surgery among stage 0-3	97.5	96.4	-1.1 (-3.1, 0.9)	97.1	96.4	-0.7 (-2.7, 1.2)	-0.4 (-3.2, 2.4)	0.80	-0.3 (-2.9, 2.4)	0.84
	Biopsy among stage T1aN0M0	9.5	15.8	6.2 (-2.8, 15.3)	10.9	9.7	-1.2 (-8, 5.6)	7.5 (-3.9, 18.8)	0.20	7.9 (-2.6, 18.5)	0.14
	Active surveillance among stage T1aN0M0	2.6	0	-2.6 (-25.6, 20.4)	3.4	0	-3.4 (-23, 16.3)	0.8 (-29.5, 31)	0.96	0.6 (-2.1, 3.3)	0.65
Bladder	Uninsured	10.3	13.9	3.6 (-2.6, 9.9)	15.7	23.6	7.9 (-0.2, 16)	-4.2 (-14.5, 6)	0.42	-4.8 (-14.3, 4.7)	0.32
	Medicaid	24.9	29.1	4.2 (-4.2, 12.5)	18.6	18.6	0 (-7.8, 7.7)	4.2 (-7.2, 15.6)	0.47	5.2 (-6.2, 16.5)	0.37
	Private	41.7	38.6	-3.1 (-12.2, 6)	41.3	30.7	-10.6 (-20, -1.2)	7.5 (-5.6, 20.6)	0.26	7.2 (-6.1, 20.5)	0.29
	Stage 0-1	39	35.4	-3.6 (-12.5, 5.4)	39.1	32.1	-7 (-16.4, 2.5)	3.4 (-9.6, 16.4)	0.61	2.5 (-10.5, 15.6)	0.70
	Resection among stage 0-1	91.5	95.8	4.3 (-5.4, 13.9)	93.7	100	6.3 (-23.4, 36)	-2 (-33.2, 29.2)	0.90	-1.9 (-11.4, 7.5)	0.69
	Radical cystectomy or trimodal therapy among stage 2-3	40.9	51.4	10.4 (-8.1, 29)	46	46.8	0.8 (-16.9, 18.5)	9.6 (-16, 35.2)	0.46	10.1 (-15.6, 35.8)	0.44
	Neoadjuvant chemotherapy among stage 2-3 who received radical cystectomy	32.4	36.8	4.4 (-22, 30.8)	29.7	15.8	-13.9 (-36, 8.1)	18.3 (-16.1, 52.8)	0.30	20.6 (-18.9, 60.1)	0.30
Prostate	Uninsured	7.5	6.2	-1.3 (-2.9, 0.2)	9.8	9.1	-0.7 (-2.7, 1.2)	-0.6 (-3.1, 1.9)	0.63	-0.6 (-3.1, 1.9)	0.66
	Medicaid	16.6	17.4	0.8 (-1.6, 3.2)	8.7	10	1.3 (-0.7, 3.3)	-0.4 (-3.6, 2.7)	0.78	-0.4 (-3.4, 2.7)	0.80
	Private	57	56.1	-0.9 (-4, 2.3)	60	57.7	-2.3 (-5.6, 1.1)	1.4 (-3.2, 6)	0.56	1.4 (-3, 5.9)	0.53
	Low risk	25.5	23.4	-2.1 (-4.8, 0.6)	27.4	27.5	0.1 (-3, 3.1)	-2.2 (-6.2, 1.9)	0.29	-2.3 (-6.4, 1.8)	0.27

© 2021 Michel KF et al. *JAMA Network Open.*

	Active Surveillance among low risk	9.8	15.6	5.8 (1, 10.5)	5.5	10.9	5.4 (1.8, 9)	0.4 (-5.6, 6.3)	0.90	0.8 (-4.4, 6)	0.76
	Prostatectomy or radiation among high risk	91.1	88.3	-2.8 (-6.1, 0.5)	92.2	90.1	-2.2 (-5.1, 0.8)	-0.6 (-5, 3.8)	0.79	0.2 (-4, 4.4)	0.93

**Models adjusted for age, race, sex, zip-code level income, metropolitan statistical area, secular year and state. Number of comorbidities and facility volume were further adjusted for treatment outcomes.*

© 2021 Michel KF et al. *JAMA Network Open.*

eTable 3. Changes in Health Insurance Coverage Between the Pre-ACA and Post-ACA Periods by Medicaid Expansion Status

		% Insurance Coverage						Model			
		Expansion States, ppt			Non-Expansion States, ppt			Crude		Adjusted[a]	
		2011-2013	2014-2016	APC (95% CI)	2011-2013	2014-2016	APC (95% CI)	DD, ppt	P	DD, ppt	P
All income combined											
Cancers Combined	Uninsured	3.9	1.6	**-2.3 (-2.5, -2.2)**	6.9	5.7	**-1.2 (-1.4, -0.9)**	**-1.2 (-1.5, -0.9)**	**<0.001**	**-1.1 (-1.4, -0.8)**	**<0.001**
	Medicaid	6.9	11.9	**5.0 (4.8, 5.3)**	5.1	5.3	0.3 (0, 0.5)	**4.8 (4.4, 5.1)**	**<0.001**	**4.5 (4.2, 4.9)**	**<0.001**
	Private	78.1	75.9	**-2.2 (-2.6, -1.8)**	74	74.4	**0.5 (0, 1)**	**-2.7 (-3.3, -2.1)**	**<0.001**	**-3.1 (-3.6, -2.5)**	**<0.001**
Kidney	Uninsured	5.7	2.2	**-3.5 (-3.8, -3.2)**	10	7.9	**-2.1 (-2.7, -1.5)**	**-1.4 (-2.1, -0.8)**	**<0.001**	**-1.3 (-1.9, -0.7)**	**<0.001**
	Medicaid	10.6	16.6	**6.0 (5.4, 6.6)**	8.1	7.7	-0.4 (-1, 0.1)	**6.4 (5.6, 7.2)**	**<0.001**	**5.9 (5.1, 6.7)**	**<0.001**
	Private	69.7	68.7	**-1.0 (-1.8, -0.2)**	64.8	67.2	**2.4 (1.5, 3.4)**	**-3.4 (-4.6, -2.2)**	**<0.001**	**-3.5 (-4.7, -2.3)**	**<0.001**
Bladder	Uninsured	7.2	2.6	**-4.6 (-5.3, -4)**	14.1	10.2	**-3.9 (-5.2, -2.6)**	-0.8 (-2.2, 0.7)	0.32	-0.7 (-2, 0.6)	0.31
	Medicaid	12.4	17.1	**4.7 (3.6, 5.8)**	10.7	9.3	**-1.4 (-2.6, -0.2)**	**6.1 (4.5, 7.7)**	**<0.001**	**5.7 (4, 7.4)**	**<0.001**
	Private	65.9	65.1	-0.7 (-2.2, 0.7)	58.7	63.7	**5 (3, 7)**	**-5.7 (-8.2, -3.3)**	**<0.001**	**-5.7 (-8.1, -3.3)**	**<0.001**
Prostate	Uninsured	2.9	1.2	**-1.7 (-1.9, -1.6)**	4.9	4	**-0.9 (-1.1, -0.6)**	**-0.9 (-1.2, -0.5)**	**<0.001**	**-0.8 (-1.1, -0.5)**	**<0.001**
	Medicaid	4.9	9.1	**4.2 (3.9, 4.5)**	3.3	3.7	**0.4 (0.1, 0.6)**	**3.8 (3.4, 4.2)**	**<0.001**	**3.7 (3.4, 4.1)**	**<0.001**
	Private	82.6	80.6	**-1.9 (-2.4, -1.5)**	79.2	79.4	0.3 (-0.3, 0.8)	**-2.2 (-2.9, -1.5)**	**<0.001**	**-2.8 (-3.4, -2.1)**	**<0.001**
Low-income											
Cancers Combined	Uninsured	8.2	2.5	**-5.7 (-6.4, -4.9)**	11.5	10.2	**-1.3 (-2.4, -0.2)**	**-4.4 (-5.7, -3)**	**<0.001**	**-4.4 (-5.7, -3)**	**<0.001**
	Medicaid	18.3	29.7	**11.4 (10, 12.9)**	11.4	11.6	0.3 (-0.9, 1.4)	**11.2 (9.3, 13)**	**<0.001**	**9.8 (8, 11.7)**	**<0.001**
	Private	52.4	48.5	**-3.8 (-5.5, -2.1)**	54.4	54.5	0.1 (-1.6, 1.9)	**-3.9 (-6.4, -1.5)**	**<0.001**	**-3.6 (-6.1, -1.2)**	**<0.001**
Kidney	Uninsured	9.9	2.9	**-7.0 (-8.5, -5.6)**	14.3	12.6	-1.7 (-3.9, 0.5)	**-5.3 (-8, -2.7)**	**<0.001**	**-5.2 (-7.8, -2.5)**	**<0.001**
	Medicaid	20.6	31.9	**11.3 (8.6, 14.1)**	15.1	12.4	-2.6 (-4.8, -0.4)	**13.9 (10.4, 17.5)**	**<0.001**	**12.4 (8.9, 16)**	**<0.001**
	Private	45.1	44.3	-0.8 (-3.9, 2.3)	46.6	49	2.4 (-0.8, 5.6)	-3.2 (-7.7, 1.3)	0.16	-2.9 (-7.4, 1.5)	0.20
Bladder	Uninsured	11.5	4.2	**-7.3 (-10.5, -4)**	18.1	15.4	-2.8 (-7.7, 2.2)	-4.5 (-10.4, 1.4)	0.13	-4.8 (-10.7, 1.1)	0.12
	Medicaid	25.2	32.6	**7.3 (1.6, 13)**	18.6	19.8	1.2 (-4, 6.4)	6.1 (-1.6, 13.8)	0.12	4.5 (-3.2, 12.2)	0.25
	Private	40.9	40.3	-0.6 (-6.7, 5.6)	38.1	41.7	3.7 (-2.8, 10.1)	-4.3 (-13.2, 4.7)	0.35	-4.0 (-12.9, 4.9)	0.38
Prostate	Uninsured	7.1	2.1	**-5 (-5.8, -4.1)**	9.6	8.3	-1.4 (-2.6, -0.1)	**-3.6 (-5.1, -2.1)**	**<0.001**	**-3.9 (-5.4, -2.3)**	**<0.001**
	Medicaid	16.5	28.2	**11.7 (9.9, 13.5)**	9.1	10.2	1.1 (-0.2, 2.4)	**10.6 (8.3, 12.8)**	**<0.001**	**9.4 (7.2, 11.6)**	**<0.001**
	Private	56.7	51.8	**-4.9 (-7.1, -2.7)**	59.4	59	-0.4 (-2.6, 1.8)	**-4.5 (-7.6, -1.4)**	**<0.001**	**-4.3 (-7.3, -1.2)**	**0.01**

[a]Models adjusted for age, race, sex, and zip-code level income, metropolitan statistical area (fixed effect), and state (random effect)

APC= Absolute Percent Change. ppt=percentage points. DD=difference-in-difference

© 2021 Michel KF et al. *JAMA Network Open.*

eTable 4. Percentages of Health Insurance Type by Medicaid Expansion Status for All-Income and Low-Income Populations by 6-Month Increments

Year	All Income						All Income					
	Expansion			Expansion			Non-expansion			Non-expansion		
	Uninsured	Uninsured	Medicaid	Private	Uninsured	Medicaid	Private	Medicaid	Private	Uninsured	Medicaid	Private
2011	3.56	3.56	6.09	79.93	6.21	4.72	75.41	6.09	79.93	6.21	4.72	75.41
	3.92	3.92	6.49	79.40	6.25	4.92	75.20	6.49	79.40	6.25	4.92	75.20
2012	3.96	3.96	7.11	78.28	7.29	4.96	73.22	7.11	78.28	7.29	4.96	73.22
	4.13	4.13	6.99	77.62	7.21	5.47	73.67	6.99	77.62	7.21	5.47	73.67
2013	4.02	4.02	7.28	77.34	7.34	5.30	73.04	7.28	77.34	7.34	5.30	73.04
	3.74	3.74	8.49	76.21	7.18	4.97	72.56	8.49	76.21	7.18	4.97	72.56
2014	2.33	2.33	9.74	77.04	6.29	5.41	73.52	9.74	77.04	6.29	5.41	73.52
	2.16	2.16	11.29	75.52	5.98	5.33	73.89	11.29	75.52	5.98	5.33	73.89
2015	1.72	1.72	11.50	75.93	5.46	5.52	74.20	11.50	75.93	5.46	5.52	74.20
	1.79	1.79	11.82	75.49	5.90	5.11	73.90	11.82	75.49	5.90	5.11	73.90
2016	1.46	1.46	11.38	75.90	5.40	5.19	75.45	11.38	75.90	5.40	5.19	75.45
	1.62	1.62	12.23	76.01	5.47	5.42	75.40	12.23	76.01	5.47	5.42	75.40

© 2021 Michel KF et al. *JAMA Network Open.*

eTable 5. Percentages of Low Risk Diagnoses for Prostate Cancer in All-Income and Low-Income Populations by 6-Month Increments

Year	All Income		Low-Income	
	Expansion	Non-expansion	Expansion	Non-expansion
2011	35.40	33.21	26.92	30.50
	32.99	32.62	26.12	25.81
2012	31.98	30.05	25.13	28.10
	30.47	29.54	23.28	24.82
2013	29.33	29.91	24.18	25.43
	28.20	28.96	22.52	30.63
2014	27.75	27.42	23.17	24.21
	26.52	27.12	21.48	22.26
2015	25.88	25.91	22.56	22.36
	25.77	24.79	22.95	20.07
2016	25.25	24.08	20.28	21.31
	25.26	22.80	21.32	18.09

© 2021 Michel KF et al. *JAMA Network Open.*

eTable 6. Percentage of Patients With Low-Risk Prostate Cancer Receiving Active Surveillance for All-Income and Low-Income Populations by 6-Month Increments

Year	All Income		Low-Income	
	Expansion	Non-expansion	Expansion	Non-expansion
2011	7.35	4.11	8.37	2.50
	8.74	5.65	10.70	5.77
2012	10.69	7.52	9.00	6.82
	12.07	6.99	10.06	8.77
2013	16.54	11.33	15.00	10.94
	16.51	11.36	19.88	10.10
2014	19.63	13.11	21.26	13.02
	20.21	13.39	22.52	11.64
2015	24.18	15.58	21.60	16.98
	26.05	17.64	23.98	11.38
2016	29.20	20.64	28.97	22.31

© 2021 Michel KF et al. *JAMA Network Open.*

eTable 7. Assessment of Robustness of Statistically Significant Associations Observed to Unmeasured Confounding

	E-value*	
	Point estimate	Lower confidence interval limit
All income combined		
Kidney cancer stage 1	3.4	1.1
NCCN "low" or "very low" risk localized prostate cancer Active Surveillance	41.3	14.3
Low-income		
Kidney cancer stage 1	68.7	1.3
Prostate cancer low risk	15.0	1.3
NCCN "low" or "very low" risk localized prostate cancer Active Surveillance	61.8	1.0

**The E-value represents the minimum strength of association, on the risk ratio scale, that an unmeasured confounder would need to have with both the treatment and outcome to fully explain away a specific treatment-outcome association, condition on the measured covariates*

© 2021 Michel KF et al. *JAMA Network Open.*

eFigure. Changes in Percentages of Health Insurance Type by Medicaid Expansion Status for All-Income and Low-Income Populations

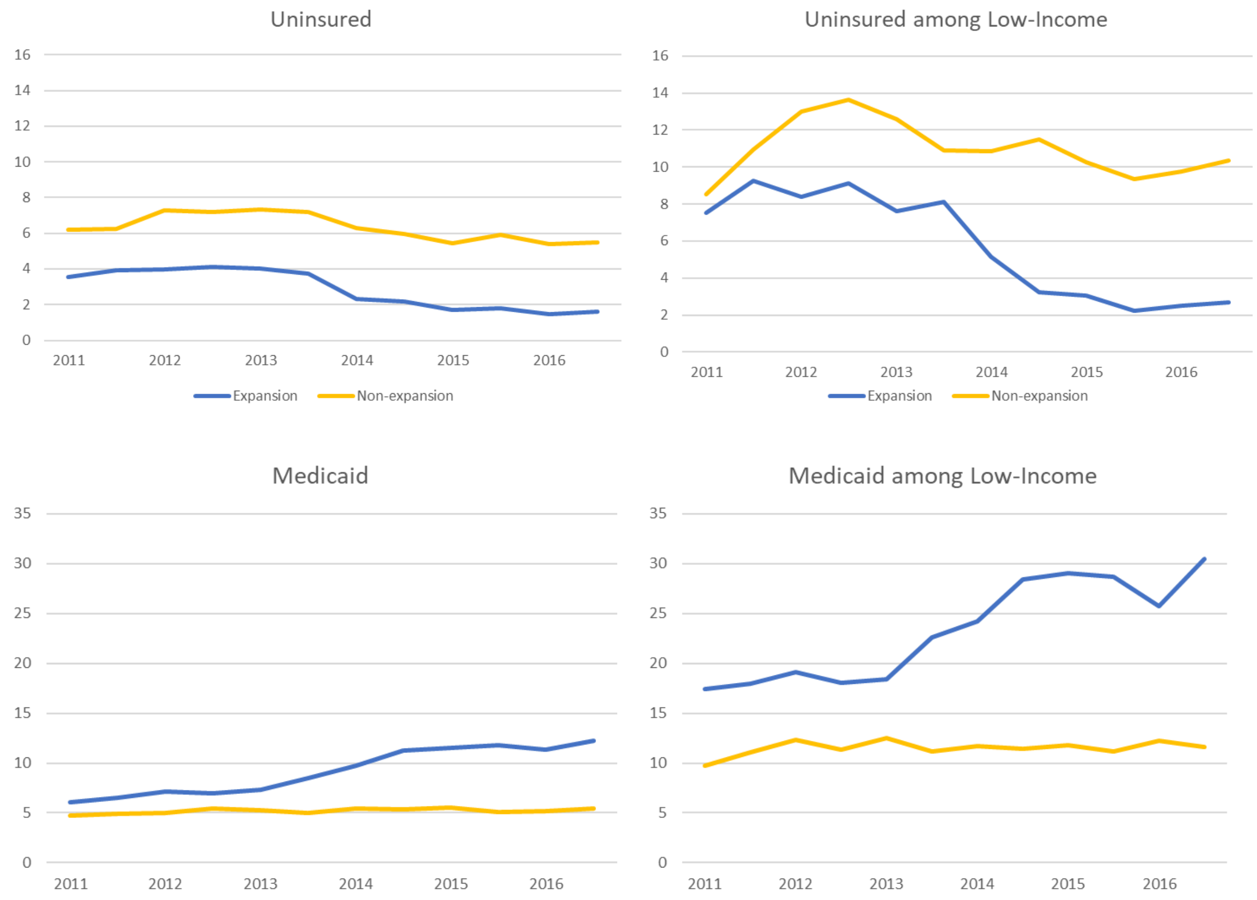

© 2021 Michel KF et al. *JAMA Network Open.*

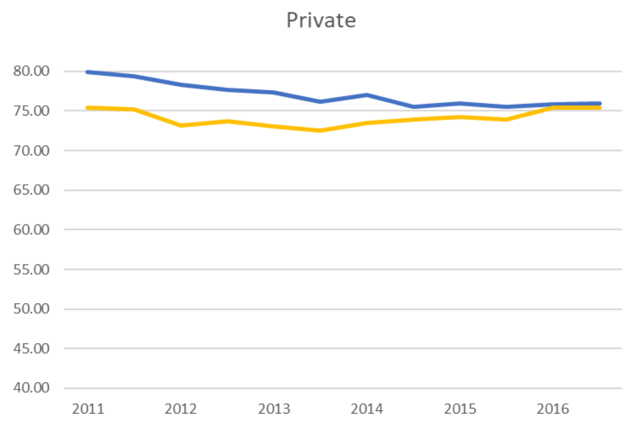

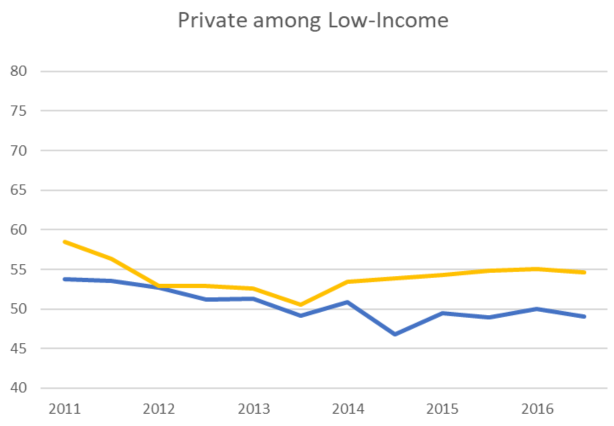

Data are shown in Supplementary Table 4. Cases in phase-in/washout period were included in the figure.

© 2021 Michel KF et al. *JAMA Network Open.*
